# Dietary patterns and risk of bladder cancer: a systematic review and meta-analysis

**DOI:** 10.1186/s12889-022-12516-2

**Published:** 2022-01-11

**Authors:** Mostafa Dianatinasab, Elaheh Forozani, Ali Akbari, Nazanin Azmi, Dariush Bastam, Mohammad Fararouei, Anke Wesselius, Maurice P. Zeegres

**Affiliations:** 1 Department of Epidemiology, Faculty of Public Health, Shiraz University of Medical Sciences, Shiraz, Iran; 2 Department of Complex Genetics and Epidemiology, School of Nutrition and Translational Research in Metabolism, Maastricht University, Maastricht, The Netherlands; 3 School of Physical and Occupational Therapy, McGill University, Montreal, Canada; 4 Graduate student and Research assistant, The college of health sciences, The University of Memphis, Memphis, USA; 5 Department of Epidemiology, Iran University of Medical Sciences, Tehran, Iran; 6 Medical School, Yasuj University of Medical Sciences, Yasuj, Iran

**Keywords:** Western Diet, Mediterranean Diet, Dietary Inflammatory Index, Bladder Cancer, Meta-analysis

## Abstract

**Background:**

Several studies have investigated the relationship between dietary patterns and the risk of bladder cancer (BC) in different regions including Europe, the United States, and Asia, with no conclusive evidence. A meta-analysis was undertaken to integrate the most recent information on the relationship between a data-driven Western diet (WD), the Mediterranean diet (MD), and dietary-inflammatory-index (DII) and the risk of BC.

**Method:**

We looked for published research into the relationship between dietary patterns and the incidence of BC in the PubMed/Medline, Cochrane Library, Web of Science, and Scopus databases up until February 2021. Using a multivariate random-effects model, we compared the highest and lowest categories of WD, MD and DII patterns and provided the relative risk (RR) or odds ratios (OR) and 95 percent confidence intervals (CIs) for the relevant relationships.

**Results:**

The analysis comprised 12 papers that were found to be suitable after scanning the databases. Both case–control (OR 0.73, 95% CI: 0.52, 0.94; I^2^ = 49.9%, *n* = 2) and cohort studies (RR 0.93, 95% CI: 0.88, 0.97; I^2^ = 63%, *n* = 4) found a substantial inverse association between MD and BC. In addition, although cohort studies (RR 1.53, 95% CI 1.37, 1.70; I^2^ = 0%, *n* = 2) showed a direct association between WD and BC, case–control studies (OR 1.33, 95% CI 0.81, 1.88; I^2^ = 68.5%, *n* = 2) did not. In cohort studies, we found no significant association between DII and BC (RR 1.02, 95% CI 0.93, 1.12; I^2^ = 38.5%, *n* = 2). In case–control studies, however, a strong direct association between DII and BC was discovered (RR 2.04, 95% CI 1.23, 2.85; I^2^ = 0%, *n* = 2).

**Conclusion:**

The current meta-analysis showed that MD and WD have protective and detrimental effects on BC risk, respectively. No significant association between DII and the risk of BC was observed. More research is still needed to confirm the findings. Additional study is warranted to better understand the etiological mechanisms underlying how different dietary patterns affect BC.

**Trial registration:**

*Protocol registration number:* CRD42020155353.

*Database for protocol registration:* The international prospective register of systematic reviews database (*PROSPERO*).

*Data of registration:* August 2020.

## Background

Being among the top ten most common types of cancer in the world, cancer of the bladder (BC) causes approximately 550,000 new cases annually [1]. With regard to the geographical distribution the risk of bladder cancer is the highest in Southern and Eastern Europe, Africa, the Middle East, and North America[2]. About 75% of cases of BC are non-muscle-invasive bladder cancer (NMIBC), a type that frequently recur and requires intensive treatment and follow-up measures posing a large burden on any national health care budgets [3]. Epidemiological studies introduced several factors that potentially influence the risk of bladder cancer. These factors include, sex, age, occupation, and smoking [3, 4]. Urinary tract infections and exposures to arsenic or aromatic amines like heterocyclic amines (HCAs), and polycyclic aromatic hydrocarbons (PAHs) are also among the potential risk factors for BC [5]. Furthermore, more information is becoming available on the possible role of food in the development of BC [5]. However, according to the latest report from World Cancer Research Fund (WCRF), the evidence from epidemiologic studies on the above association is scarce and largely inconsistent [6].

Epidemiological studies suggested that several environmental and lifestyle related factors, e.g., pollutions and diet, might also play important roles in the risk of BC [7, 8]. In terms of diet, epidemiological studies have examined at the associations between certain foods and the risk of BC, with some intriguing results. As such, animal fat, a high red meat intake, and refined carbohydrate, that are the major component of the Western diet (WD), are associated to an elevated risk of BC [9–11]. In contrast, the Mediterranean diet's key components, fruits, vegetables, whole grains, and dietary fiber, have been associated to a lower incidence of BC [12–17]. The MD contains sufficient of fiber (found in fruits and vegetables), legumes and grains, fish, moderate wine intake, low-to-moderate milk and dairy products consumption, and minimal meat and meat products consumption [16, 18]. WD, on the other hand, is a dietary pattern that includes a lot of high-fat animal meat, processed products, red meat, and high-sugar foods [19–21]. Based on the existing evidence, MD is a significant protective factor for several non-communicable diseases [22–24].

Foods contain many interacting nutrients affecting body’s function and well-being. Although several studies associated particular food items are with BC, the evidence is inconclusive [25, 26]. This is because, individuals do consume food items together and it is therefore rather than focusing at individual nutrients when analyzing food, it's critical to apply a holistic approach. Among the several methods in nutritional epidemiology, dietary pattern analysis is now often regarded as a more effective method for determining the overall impact of food consumption on health. Given the fact that the relationship between dietary pattern and BC has attained increasing attention, the evidence remains inconclusive. For example, a few studies reported hazardous effects of WD on the risk of BC [9–11], whereas others found an inverse association between WD (or healthy diets) and BC [12–17]. To sum up, although the association of BC in association to dietary pattern, has been investigated by several researchers in Europe, United States, and Asia, no conclusive evidence over the subject has been made. We performed a meta-analysis of cohort and case–control studies to integrate the most recent evidence on the relationship between WD, MD, and DII and the risk of BC among those who were suffering from the BC.

## Methods

This study was carried out in accordance with the preferred reporting items for systematic reviews and meta-analyses (PRISMA) standard recommendations [27].

### Protocol and registration

The aim of this study was to see if there was an association between dietary habits and the risk of developing BC. In August 2020, the study protocol was registered with the CRD42020155353 registration number in the international prospective register of systematic reviews database (PROSPERO) (Available at: https://www.crd.york.ac.uk/PROSPERO/display_record.php?RecordID=155353).

### Search strategy and selection criteria

Without restrictions, we searched PubMed/Medline, Web of Science (ISI), Cochrane library, Clinicaltrials.gov, and SCOPUS databases for papers that indicated a relation between dietary patterns and the risk of BC up to February 2021. The following search keywords or phrases were used to find relevant articles: ("neoplasm" OR "cancer" OR "carcinoma") AND ("bladder" OR "urinary bladder") AND ("dietary pattern" OR "eating pattern" OR "food pattern" OR "dietary habit" OR "diet" OR "dietary"). Additionally, the reference lists of the included papers and recent major reviews were carefully evaluated to find other relevant publications in order to prevent missing any related article. Review studies, and if the retrieved publications didn’t fulfilled the following inclusion criteria, they were excluded in our study: studies with a case–control or cohort design, reported the associations between dietary patterns and BC, included newly diagnosed cases of BC, diagnosed all cases using pathological biopsies or other standard methods, and provided relative risks (RRs), hazards ratios (HRs), or odds ratios (ORs) and their corresponding 95 percent confidence intervals for the dietary patterns. We included the most often identified dietary patterns across studies to reduce the possibility of misclassifications, and we made sure that the selected dietary patterns were specified consistently in terms of factor loadings of the most frequently consumed foods as much as feasible. The categorization of Western, Mediterranean and DII dietary patterns was based on selected peer-reviewed publications. When several publications from the same data were found, the publication with the most participants/person-years was chosen. The selected articles and reading the titles and abstracts of the searched papers independently were examined by two independent reviewers (NA and DB). If both reviewers agreed that a publication did not fulfill the above-mentioned inclusion criteria, it was excluded. Inconsistencies (if any) were to be solved by a consultation with a third author (MD).

### Data extraction and quality assessment

Using a standardized data collection form, two reviewers independently extracted the required information. From each study, we gathered the following data: first author's last name; year of publication; study location; study design; sample size; duration of follow-up; method of analysis; diagnostic criteria; gender; average age of participants; dietary valuation methods; dietary patterns; RRs, HRs, or ORs and the corresponding 95% CIs for the highest vs. the lowest categories; of dietary patterns from the final adjusted models and potential confounders adjusted in the multivariate analysis. The authors were contacted by email at least twice, one week apart, when the full text of a paper was unavailable or if any essential information was missing in the provided data. The Newcastle–Ottawa Scale (NOS) was used to measure quality assessment of the included studies [28]. Concisely, we used a nine-score tool based on the NOS to assess the quality of the studies characterized by three broad criteria: [1] appropriate study population selection, [2] study group comparability, and [3] ascertainment of the exposure (for cohort studies) or outcome (for case control studies) of interest. Each study's quality was independently assessed by two reviewers (NA and DB). Disagreements were once again resolved by discussion among the reviewers. Studies having a score of 7 or above, with 9 being the maximum, were deemed to be of high quality.

### Statistical analyses

The observed relationship between dietary patterns and the risk of BC was measured using RRs as the common scale. As RR estimators, HRs, ORs, and incidence rate ratios (IRRs) were also utilized [29]. We conducted random-effects meta-analysis to obtain the pooled RR and its 95% confidence intervals.

Because of the potential heterogeneity in clinical and methodological characteristics within and between studies, the random-effects analysis was used [30].

To assess heterogeneity across studies, we utilized Q statistics with a significance level of *P* < 0.10. We also used the I^2^ statistic to indicate the variance between studies that may be attributed to heterogeneity rather than chance. Moderate heterogeneity was defined as an I^2^ value larger than 50% [31].

To measure the impact of individual or a group of studies on the results e conducted a sensitivity analysis. We tested for publication bias by visual inspection of Begg’s funnel plots presenting log RRs against their standard errors (SEs) [32, 33]. STATA version 15.0 was used for all analyses (Stata Corp LP, College Station, Texas). Except otherwise specified, statistical significance was defined as a P-value of less than 0.05.

## Results

### Study characteristics

**Fig. 1:**
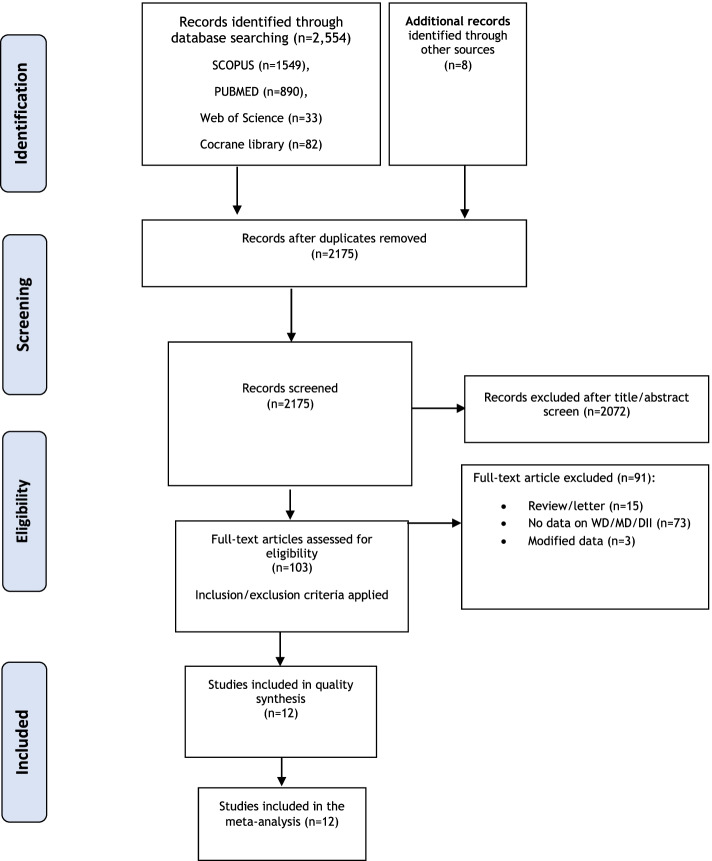
PRISMA flow diagram of the study selection process

Following the PRISMA flow diagram (**Fig. ****1**) of the study selection process, we found a total of 2554 articles from the searched databases. Some were excluded because of duplication and being irrelevant articles. Eventually, seven cohort studies [10, 11, 14–17, 34], and five case control studies [9, 12, 13, 35, 36] were included in the present mete-analysis. Included cohort studies consisted of 12,679 cases and 1,952,859 non-cases. In addition, the case–control studies included 1891 cases and 2326 controls. The study selection procedure is illustrated in Fig. 1.

The details of the included studies are shown in Table 1. Of the Included articles that were published between 2008 and 2020, six studies assessed the effect of MD on BC risk [12–17], three articles investigated the associations between WD and BC [9–11], and three studied on DII and BC [34–36]. Two of them were conducted in Italy [12, 35] and others were conducted in Netherlands [15], two from EPIC study [14, 16], Belgium [13], Australia [17], Uruguay[9], Iran [36], united states [11, 34], and one from Australia, European countries and united states [10]. Dietary intake was assessed using food-frequency questionnaire (FFQ) in almost all the included studies. Adjustment-variables were mostly age, sex, smoking, total energy intake, body mass index, alcohol consumption, physical activity, and family history of BC.

**Table 1:** The characteristics of the included studies in the meta-analysis

Author	Year	Location	Study design	Sex (n%)	Follow up duration	Sample size and characteristics	Mean Age	Method of analysis	invasive or non-invasive	diet components	Dietary patterns investigated and associated risk
**Schulpen, et al**	2019	Netherlands	Cohort	Men 48% Women 52%	20.3 years	2049 cases 4,084 sub cohort members	55–69	Trichopoulou	996 invasive/1053 non-invasive	Proxy of MD: vegetables, legumes, fruits, nuts, whole grains, fish, the ratio of MUFA to saturated fatty acids	MD (HR = 1.00, 95% CI:0.92,1.09) total
**Witlox, et al**	2020	European Countries	Cohort	Men 47% Women 53%	6,577,179 person years	3639 cases/642,583 non-case	younger than 70 years	Trichopoulou	1480 non-invasive/945 invasive	fruits, vegetables, legumes and cereals, moderate-to-high consumption of fish, moderate consumption of alcohol (mostly wine), low-to-moderate consumption of milk and dairy products, and low consumption of meat and meat products	MD (HR = 0.85,95% CI: 0.77, 0.93)
**Bravi, et al**	2018	Italy	Case–control	Men 85% Women 15%	NA	690 cases/665 controls	25–84	Trichopoulou	268 non-invasive/ 192 pT1/ 159 invasive/ 307 moderately or well differentiated/ 312undifferentiated or poorly differentiated	olive oil, fruits, vegetables, legumes, and whole grain cereals	MD (OR = 0.66,95% CI:0.47–0.93)
**Buckland, et al**	2014	EPIC	Cohort	Men 30% Women 70%	11 years	1575 cases 475,737 non cases	51.2 6 ± 9.9	Trichopoulou	430 were aggressive and 413 were non-aggressive UCC tumors and for 582 subject’s tumor aggressiveness was unknown (n 5 52) or not validated (n 5 530)	fruit, nuts and seeds, vegetables, legumes, fish, olive oil and cereals (dairy products and meat, calculated as a function of energy)	MD (HR = 0.84, 95% CI: 0.69, 1.03)
**Brinkman, et al**	2011	Belgium	Case–control	Men69% Women 31%	NA	200 cases/386 controls	cases 67.6 ± 9.9 controls 64.2 ± 9.6	PCA	no data	dietary fat, meat, olive oil, fish, eggs, milk, cheese, margarine	WD (OR: 1.11, 95% CI:0.67–1.83)
Dugué, et al	2016	Australia	Cohort	Men 41% Women 59%	21.3 years	379 Cases/37063 Non-cases	27 to 76	Trichopoulou	165 invasive/ 214 superficial	MD: vegetables, fruits, cereals, legumes, and fish	MD:( HR = 0.97, 95% CI: 0.88–1.08
**Dianatinasab, et al**	2020	Australia, European Countries and united states	Cohort	Men 33% Women 67%	11.4 years	3401cases /577 367 non-cases	52.7 years (± 10.2) for cases and 60.5 (± 7.3) 52.6 (± 10.1) for controls	priori	1365 no muscle-invasive / 874 muscle-invasive	Cream, Egg, Red and processed meet, Butter, Margarine, Animal fat, Pasta, Sugar, Dressing, Dips, Vegetables, Fruits, Fluid	WD (HR = 1.54, 95% CI: 1.37–1.72)
**Westhoff, et al**	2018	Texas	Cohort	Men 80% Women 20%	median of 65.7 months	595 case	no restrictions on age	factor analysis	only 595 non-invasive selected then 120 progressed to muscle-invasive bladder cancer during study	western: Cornbread, Black eyed peas, Fried chicken, Fried fish, Okra, Gravy, Canned chili, green beans, French fries, bacon, corn, hamburgers, beef, pork, potato, sausages, wine/ fruit and vegetables	WD (HR = 1.48,95% CI:1.06–2.06)
**Stefani, et al**	2008	Uruguay	Case–control	Men 88% Women 12%	NA	255 cases/501 controls	30–89	factor analysis	no data	sweet beverage: coffee, tea, and added sugar/western patter: red meat, fried eggs, potatoes, and red wine/prudent pattern: fresh vegetables, cooked vegetables, and fruits	WD (OR = 2.35, 95% CI 1.42–3.89 MD (OR = 1.06, 95% CI 0.67–1.68)
**Shivappa, et al**	2019	Iran	Case–control	Men 92% Women 8%	NA	56 cases/109 controls	48–73	Multivariate analyses	no data	bread, rice, meat, fish and	Dietary inflammatory index (DII) score > –0.12 (OR = 2.46; 95% CI:1.12–5.41) among current/ex-smokers (OR DII (> –0.12/ –0.12) 3.30; 95% CI¼1.07–10.16
**Abufaraj, et al**	2019	United States	Cohort	Men 20% Women 80%	23 years	1,042 cases/ 218,074 non-case	25–75	EDIP score assessment	no data	red meat, processed meat, all vegetables, fish, high energy beverages, carbonated beverages, low energy beverages, tomatoes, beer; wine; tea; coffee; dark yellow vegetables, snacks; fruit juice; and pizza	DII (RR = 0.92, 95% CI: 0.75–1.12)
**Shivappa, et al**	2017	Italy	Case–control	Men 84% Women 16%	NA	690 cases/665 controls	25–80	factor analysis	460 noninvasive/159 invasive/ 307 moderately or well differentiated/ 312undifferentiated or poorly differentiated	carbohydrates, proteins, fats, alcohol, fibers, cholesterol, saturated fatty acids, monounsaturated fatty acids, polyunsaturated fatty acids, omega 3, omega 6, niacin, thiamin, riboflavin, vitamin B6, iron, zinc, vitamin A, vitamin C, vitamin D, vitamin E, folic acid, beta carotene, anthocyanidins, flavan3ols, flavonols, flavanones, flavones, isoflavones, caffeine, and tea	DII (OR Continuous = 1.11, 95% CI = 1.03, 1.20)/ (OR Quartile4vs1 = 1.97, 95% CI = 1.28, 3.03)
**Author**		**Year**		**Events followed**		**Diagnostic criteria**		**MD/WD compliance assessment method**		**Variables for adjustment**	
**Schulpen, et al**		2019		Bladder Cancer Risk		record linkage with the Netherlands cancer Registry and the nationwide Dutch Pathology Registry		FFQ		age, sex	
**Witlox, et al**		2020		Bladder Cancer Risk		pathology confirmed cases		FFQ		sex, age, smoking, total energy intake	
**Bravi, et al**		2018		Bladder Cancer Risk		incident diagnosis of urothelial carcinoma of the bladder (93%histologically confirmed)		FFQ		Age, sex, BMI, study center, year of interview, Education, Smoking, non-alcohol energy intake, History of Diabetes, History of Cystitis, Family history of bladder cancer	
**Buckland, et al**		2014		Bladder Cancer Risk		All newly diagnosed by pathology reports		dietary questionnaires		smoking, dietary energy	
**Brinkman, et al**		2011		Bladder Cancer Risk		histologically confirmed with transitional cell carcinoma		FFQ		age, sex, smoking characteristics, occupational exposures, calorie intake	
Dugué, et al		2016		Bladder Cancer Risk		identified from Victorian cancer registry and the Australian Cancer Database		FFQ		sex, country of birth, smoking, alcohol consumption, body mass index physical activity, education, and socioeconomic status	
**Dianatinasab, et al**		2020		Bladder Cancer Risk		the International Classification of Diseases for Oncology (ICD-O-3 code C67) using population-based cancer registries, health insurance records or medical records		FFQ		total energy intake in kilocalories, sex, smoking status (never, former or current smoker) and smoking intensity, fluid, vegetables and fruits intake	
**Westhoff, et al**		2018		risk of recurrence and progression in non- muscle-invasive bladder cancer		newly histologically confirmed NMIBC		FFQ		age, sex, education, income, body mass index, smoking status and intensity, total energy intake, grade, tumor multiplicity, concomi- tant carcinoma in situ, and treatment	
**Stefani, et al**		2008		Bladder Cancer Risk		newly diagnosed and micro- scopically confirmed cases of transitional cell carcinoma of the bladder with hospitalized controls		FFQ		age, sex, residence, urban/rural status, education, family history of bladder cancer, high-risk occupation, body mass index, years smoked, and total energy intake	
**Shivappa, et al**		2019		Bladder Cancer Risk		histologically confirmed cases		FFQ		age, sex, body mass index (BMI), physical activity, smoking status, alcohol use and family history of cancer	
**Abufaraj, et al**		2019		Bladder Cancer Risk		confirmed by retrieving relevant medical records		FFQ		age, energy intake, smoking status, fluid intake, nonsteroidal anti- inflammatory drug use, pregnancy, menopausal status, age at menopause	
**Shivappa, et al**		2017		Bladder Cancer Risk		histologically confirmed cases of BC		FFQ		age, sex, year of interview, study center, and total energy intake, education, smoking	

#### Association between a Western dietary patterns and risk of BC

**Fig. 2:**
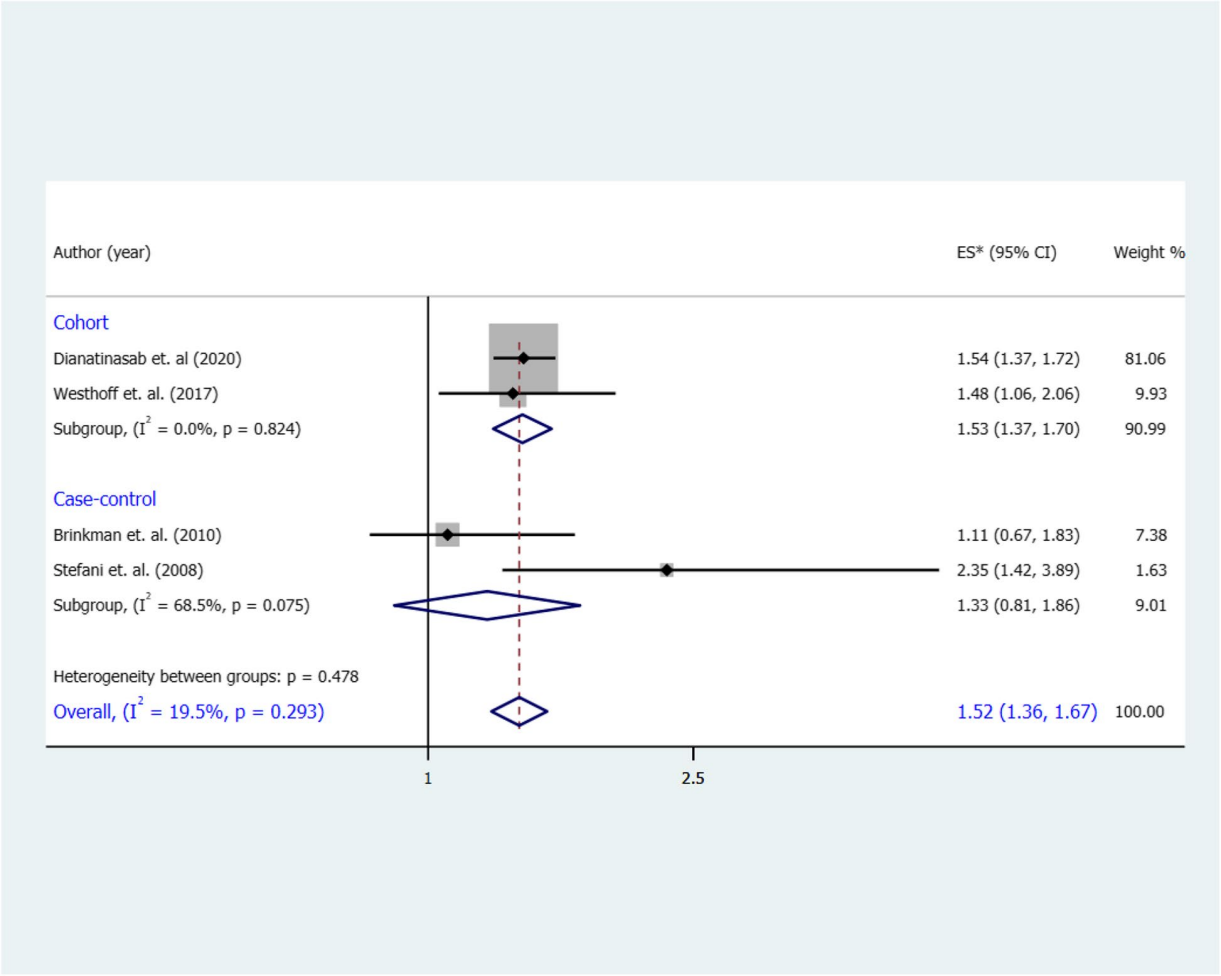
Forest plot shows the association between the highest category of a WD and BC risk

The combined RR for the highest vs. the lowest category of a WD and risk of BC was 1.52 (95% CI 1.36, 1.67), with no significant heterogeneity (I^2^ = 19.5%, *p* = 0.29) (**Fig. ****2**). A similar pattern of association was observed in cohort studies (RR 1.53, 95% CI 1.37, 1.70), again with no heterogeneity (I^2^ = 0%, *p* = 0.82). In contrast, we found no significant association between a WD and risk of BC in case–control studies (OR 1.33, 95% CI 0.81, 1.88; I^2 ^= 68.5%, *p* = 0.07).

#### Association between Mediterranean diet and risk of BC

**Fig. 3:**
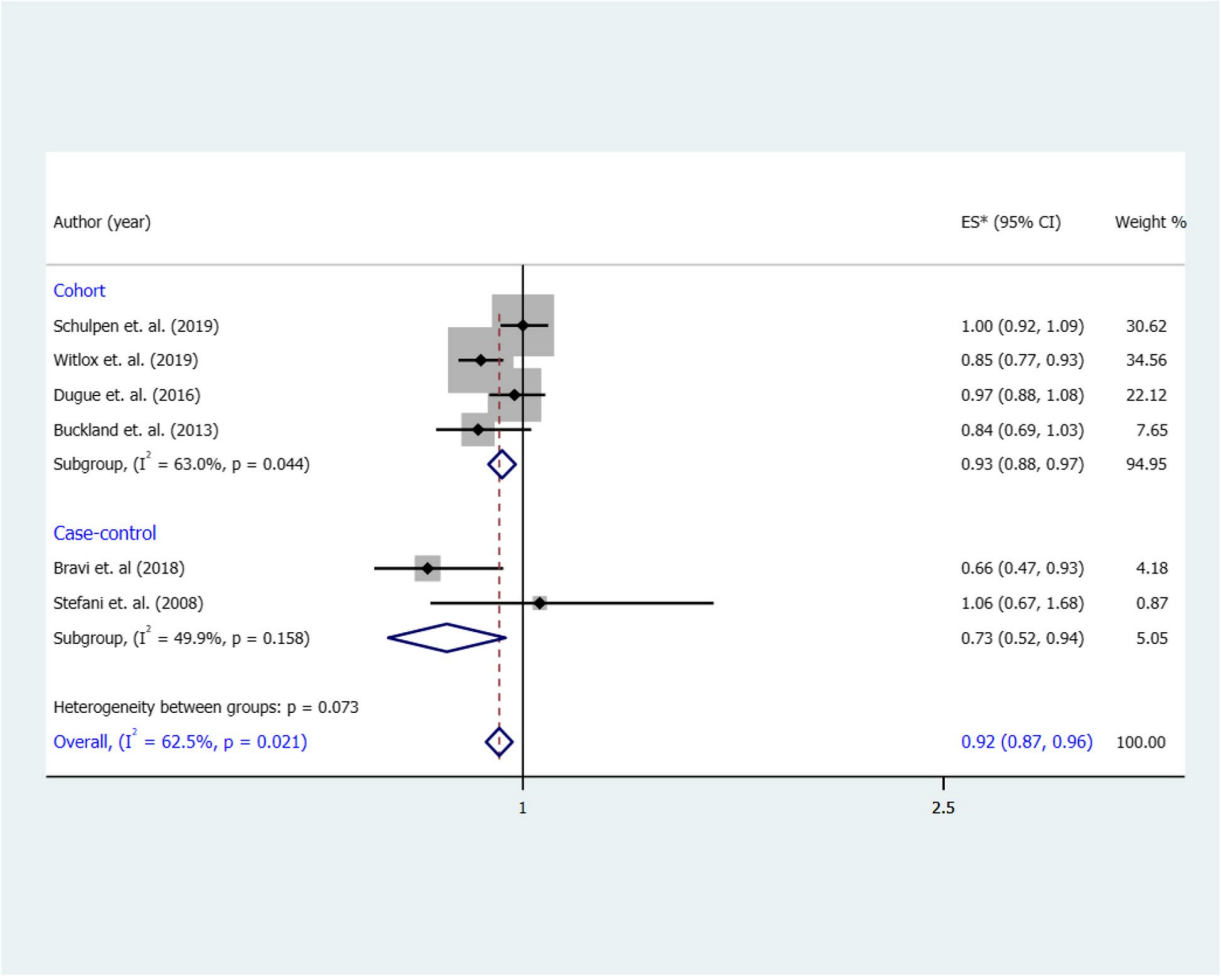
Forest plot shows the association between the highest category of a MD and BC risk

According to **Fig. ****3**, six studies (4 cohorts; 2 case–control) examined the effects of a MD and risk of BC, and their results were conflicting. As shown in **Fig. ****3**, the overall RR of the association between risk of BC for the highest vs. the lowest category of MD was protective (RR 0.92, 95% CI: 0.87, 0.96), with a significant heterogeneity (I^2^ = 62.5%, *p* = 0.02). We found the same pattern with pooled estimate, in both cohorts (RR 0.93, 95% CI: 0.88, 0.97; I^2^ = 63%, *p* = 0.04) and case control studies (OR 0.73, 95% CI: 0.52, 0.94; I^2^ = 49.9%, *p* = 0.15).

#### Association between DII and risk of BC

**Fig. 4:**
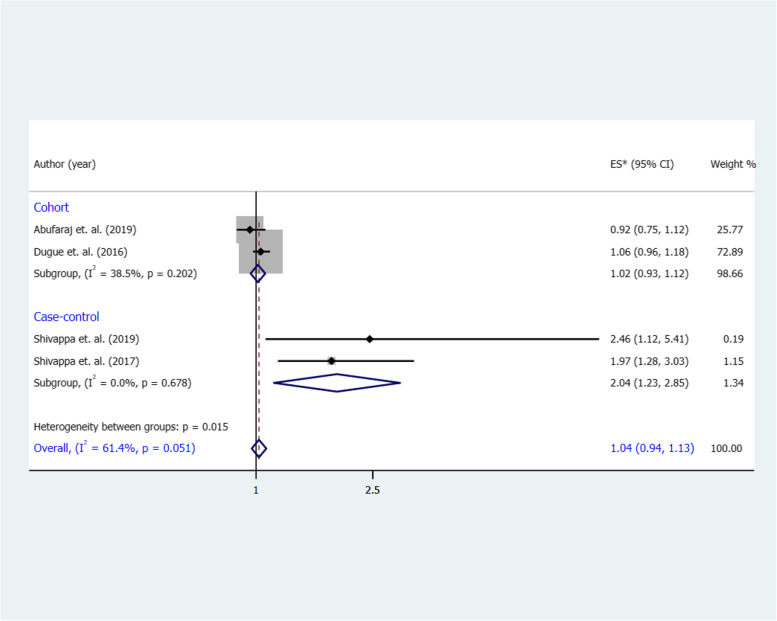
Forest plot shows the association between the highest category of a DII and BC risk

The combined RR for the highest vs. the lowest category of a DII and risk of BC was 1.04 (95% CI 0.94, 1.13), with a significant heterogeneity (I^2^ = 61.4%, *p* = 0.05) (**Fig. ****4**). We found a similar association in cohort studies (RR 1.02, 95% CI 0.93, 1.12), with no significant heterogeneity (I^2^ = 38.5%, *p* = 0.20). In case–control studies, however, a strong direct association was identified between a DII and the risk of BC (OR 2.04, 95% CI 1.23, 2.85; I^2^ = 0%, *p* = 0.67).

#### Quality assessment and sensitivity analysis

Table 2 shows the methodological quality of the selected studies according to the NOS. The NOS scores for the included studies ranged from 6 to 8, with 11 high [9, 10, 12–17, 34–36] and one medium-quality [11]. We conducted a sensitivity analysis to check if the results would change when each individual study was removed at a time. Except for studies on DII and risk of BC, the results were fairly robust after removing studies from the meta-analyses. Results of publication bias were not provided according to the reviewers suggestions.

**Table 2:** Results of the Newcastle–Ottawa Scale (NOS) for assessing the quality of case–control and cohort studies in the meta-analyses

Case–control studies	Selection				Comparability	Exposure				
	*Case* *definition*	*Representativeness of the cases*	*Selection of Controls*	*Definition of Controls*	*Control for most important factor and Control for any additional factor*	*Ascertainment of exposure*	*Same method of ascertainment for cases and controls*		*Non-Response rate*	*Total score*
Bravi 2018 [12]	(1)	(1)	(0)	(1)	(2)	(0)	(1)		(1)	7
Brinkman 2011 [13]	(1)	(1)	(1)	(1)	(2)	(0)	(1)		(1)	8
Shivappa 2017 [35]	(1)	(1)	(0)	(1)	(2)	(0)	(1)		(1)	7
Shivappa 2019 [36]	(1)	(1)	(0)	(1)	(2)	(0)	(1)		(1)	7
Stefani 2008 [9]	(1)	(1)	(0)	(1)	(2)	(0)	(1)		(1)	7
Cohort studies	Selection			Comparability		Outcome				
	*Representativeness of the exposed cohort*	*Selection of the non-exposed cohort*	*Ascertainment of exposure*	*Outcome was not present as baseline*	*Control for most important factor and Control for any additional factor*	*Assessment of outcome*	*Adequate follow-up period for outcome*	*Adequacy of follow up of cohorts*		*Total score*
Abufaraj 2019 [34]	(1)	(1)	(0)	(1)	(2)	(0)	(1)	(1)		7
Buckland 2014	(1)	(1)	(0)	(1)	(2)	(1)	(1)	(1)		8
Dianatinasab 2020 [10]	(1)	(1)	(1)	(1)	(1)	(1)	(1)	(1)		8
Dugu 2016 [17]	(1)	(1)	(0)	(0)	(2)	(1)	(1)	(1)		7
Schulpen 2019 [15]	(1)	(1)	(0)	(1)	(2)	(1)	(1)	(1)		8
Westhoff 2018 [11]	(1)	(1)	(0)	(0)	(2)	(0)	(1)	(1)		6
Witlox 2020 [16]	(1)	(1)	(1)	(1)	(1)	(1)	(1)	(1)		8

## Discussion

In the meta-analysis, we reviewed the investigated associations between adherence to major dietary patterns and risk of BC. We observed a direct association between WD and risk of BC, and an inverse association between MD and risk of developing BC. However, there was no association between DII and BC risk.

Several systematic review and meta-analyses have investigated the association between dietary patterns and the risk of cancer of other organs, WD was associated with increased risk of colorectal [37, 38], stomach [39], and prostate cancers [40]. Similar to our results, a meta-analysis with 12 observational studies reported that WD is related to an increased risk of prostate cancer but no association between healthy pattern and prostate cancer risk [40]. However, to date no meta-analysis is available on the association between dietary patterns and BC. The results published from studies that have examined the relationship between WD and risk of BC are in accordance with our findings [9–11]. For example, the results of a recently published pooled analysis on 13 cohorts suggested that adherence to a WD pattern is associated with an increased risk of BC [10]. Also, Westhoff et. al. found that greater adherence to a WD was associated with a higher risk of BC recurrence [11]. This finding supports the hypothesis that WD plays a role in the etiology and prognosis of BC. According to the results, although a strong association was observed between higher adherence to a WD and BC in cohort studies (RR 1.55, 95%CI: 1.37 to 1.70), we found no significant association between WD and risk of BC in case–control studies (RR 1.30, 95%CI: 0.81 to 1.88). This might be due to recall bias in these studies and even small sample size of the included case control studies.

Epidemiological studies have concentrated on some key elements of WD and reported a positive associations between red and processed meat, refined grain and saturated fats and risk of BC [41]. Red and processed meat is one of the important key elements of this dietary pattern and it is positively associated with the risk of BC [42]. Potentially hazardous materials present in the WD, such as N-nitroso-compounds, heterocyclic aromatic amines and polycyclic aromatic hydrocarbons in red meat, are excreted in the urine. As a result, they come into direct contact with the inner lining of the bladder wall, potentially causing cancer in urothelial cells [43]. Moreover, it is suggested that red and processed meats contain saturated fat and heme iron, potential inducers of oxidative stress and DNA damage [44]. Also, more mutagenic substitutes during the cooking procedure of these nutrients takes place. As mentioned by Matteo et. al., cooking meat or fats, main components of WD, at higher temperatures (roasting) or for prolonged times (e.g., stewing) were associated with an increased BC risk [45]. According to the previous studies, components produced during food processing, particularly when meat is cooked at higher temperatures or for longer periods of time, can damage DNA and increase the risk of cancer [45–47]. However, the lack of information on cooking and preparing food in the included studies prevented us to conduct a subgroup analysis according to the cooking methods.

Regarding adherence to MD and cancer risk, results of a systematic review reported that MD was inversely associated with cancer mortality and risk of colorectal, breast, gastric, liver, head and neck, gallbladder, and biliary tract cancers [48]. However, a meta-analysis of 10 epidemiological studies provided evidence that MD is not related with prostate cancer risk [49]. In our meta-analysis the association between MD and risk of BC was reported by 6 studies [12–17]. We found a stronger association between MD and BC in cohort studies rather than case–control studies. A pooled analysis of 13 cohort studies showed that adherence to the MD was associated with a reduced risk of developing BC (HR: 0.85; 95% CI: 0.77, 0.93), suggesting a positive effect of a MD on BC risk [16]. In addition, Dugué et al. discovered a moderate inverse relationship between MD adherence and urothelial cell cancer [17]. Also, Buckland et al. found an inverse associations between adherence to the MD and occurrence of overall, aggressive or non-aggressive, BC for both gender [14]. It is suggested that, among key elements of this diet, some of them had beneficial effects on the prevention of BC. For example, it has been shown that the consumption of vegetables and fruits, as the main components of the WD, are inversely associated with the risk of BC [50, 51]. It is suggested that, polyphenols, carotenoids, and vitamins C and E are abundant in both vegetables and fruits, and they serve as antioxidants, preventing DNA damage by neutralizing reactive oxygen species [52]. Olive oil is another significant component of the MD that has been examined as a single dietary item in relation to bladder cancer. Brinkman et al. showed that a higher consumption of olive oil was inversely related to the risk of BC [13].

Regarding DII, A meta-analysis found that higher pro-inflammatory diets are linked to an increased risk of prostate, kidney, and bladder cancer [53], results that are different with our finding. In this study, we investigated 2 case–control and 2 cohort studies [17, 34–36] on the association of DII and BC. Our pooled estimates show that DII was not significantly associated with the BC risk. Null association between a DII and BC in cohort studies suggests that the significant association found in case–control studies may be due to recall bias rather than a real association. The discrepancies between the individual studies could be attributed to the small sample sizes, study design or population substructure. Chronic inflammation causes oxidative and nitrative DNA damage in stem cells, which might be one of the processes behind the observed positive relationship between DII and BC [54].

There are probably differences in the definitions of diets in different studies, so we used the most common definition. However, there are some limitations to this meta-analysis, as such, the results are combined from studies conducted with different methods in different populations, resulting in heterogeneity. Among several potential explanations, recall bias occurs a lot in case control studies rather than cohort studies. Moreover, a possible misclassification within the considered dietary patterns may existed. We cannot generalize our results to the whole world because the most studies that we found were from European and developed countries. As a result, more studies are needed, especially in Asian and African countries, to support these findings.

## Conclusions

Our results specified a direct association between WD and risk of BC, and an inverse association between MD and risk of developing BC. Also, there was no association between DII and BC risk. According to our findings dietary patterns might play an important role in BC prevention and guidelines might provide more attention to recommend consuming MD components and reducing WD components. However, further researches are needed to confirm our findings and to study the possible mechanisms for the WD effects on carcinogenesis of BC and MD and their effects on BC prevention.

## Data Availability

The datasets used and analyzed during the current study are available from the corresponding author on reasonable request.

## Abbreviations

BC: Bladder cancer
DII: Dietary-inflammatory-index
WD: Western diet
MD: Mediterranean diet
FFQ: Food-frequency questionnaire
RRs: Relative risks
HRs: Hazard ratios
ORs: Odds ratios
Cis: Confidence intervals
SEs: Standard errors
NOS: Newcastle–Ottawa Scale
